# Human Induced Pluripotent Stem Cell-Derived Models to Investigate Human Cytomegalovirus Infection in Neural Cells

**DOI:** 10.1371/journal.pone.0049700

**Published:** 2012-11-27

**Authors:** Leonardo D'Aiuto, Roberto Di Maio, Brianna Heath, Giorgio Raimondi, Jadranka Milosevic, Annie M. Watson, Mikhil Bamne, W. Tony Parks, Lei Yang, Bo Lin, Toshio Miki, Jocelyn Danielle Mich-Basso, Ravit Arav-Boger, Etienne Sibille, Sarven Sabunciyan, Robert Yolken, Vishwajit Nimgaonkar

**Affiliations:** 1 Western Psychiatric Institute and Clinic, Department of Psychiatry, University of Pittsburgh School of Medicine, Pittsburgh, Pennsylvania, United States of America; 2 Pittsburgh Institute for Neurodegenerative Diseases and Department of Neurology, University of Pittsburgh, Pittsburgh, Pennsylvania, United States of America; 3 Ri.MED Foundation, Palermo, Italy; 4 Department of Human Genetics, Graduate School of Public Health, University of Pittsburgh, Pittsburgh, Pennsylvania, United States of America; 5 Department of Immunology, Starzl Institute, University of Pittsburgh, Pittsburgh, Pennsylvania, United States of America; 6 Division of Pulmonary, Allergy and Critical Care Medicine, University of Pittsburgh School of Medicine, Pittsburgh, Pennsylvania, United States of America; 7 Department of Pathology, University of Pittsburgh School of Medicine, Pittsburgh, Pennsylvania, United States of America; 8 Department of Development Biology, University of Pittsburgh School of Medicine, Pittsburgh, Pennsylvania, United States of America; 9 Department of Pediatrics, Cancer Research Building I, Johns Hopkins School of Medicine, Baltimore, Maryland, United States of America; 10 Stanley Division of Developmental Neurovirology, Johns Hopkins School of Medicine, Baltimore, Maryland, United States of America; University of Nebraska Medical Center, United States of America

## Abstract

Human cytomegalovirus (HCMV) infection is one of the leading prenatal causes of congenital mental retardation and deformities world-wide. Access to cultured human neuronal lineages, necessary to understand the species specific pathogenic effects of HCMV, has been limited by difficulties in sustaining primary human neuronal cultures. Human induced pluripotent stem (iPS) cells now provide an opportunity for such research. We derived iPS cells from human adult fibroblasts and induced neural lineages to investigate their susceptibility to infection with HCMV strain Ad169. Analysis of iPS cells, iPS-derived neural stem cells (NSCs), neural progenitor cells (NPCs) and neurons suggests that (i) iPS cells are not permissive to HCMV infection, i.e., they do not permit a full viral replication cycle; (ii) Neural stem cells have impaired differentiation when infected by HCMV; (iii) NPCs are fully permissive for HCMV infection; altered expression of genes related to neural metabolism or neuronal differentiation is also observed; (iv) most iPS-derived neurons are not permissive to HCMV infection; and (v) infected neurons have impaired calcium influx in response to glutamate.

## Introduction

Human cytomegalovirus (HCMV), a member of the alpha herpes viridae family, is a major cause of neurological deficits in newborns. Approximately 0.2–2% of newborns are infected prenatally and 10–15% of the infected neonates suffer from severe neurological abnormalities including microcephaly, mental retardation, and ophthalmologic abnormalities [1]. Many investigators have used rodent cells to study the relative susceptibility of different neural lineages to mouse cytomegalovirus (MCMV) infection [2], [3]. The effects of the infection vary with the model utilized. Early stage mouse embryos are not permissive for MCMV infection; i.e., even if HCMV enters the host cell, it is unable to complete replication cycles [4]. Mouse embryos infected prior to implantation develop normally to the blastocyst stage [5]. If mouse embryos are infected with MCMV at mid-gestation, viral-susceptible cells are first detected in the placenta, followed by blood cells, endothelial and mesodermal cells. In rodent brains, MCMV localizes to the ventricular and sub-ventricular zone, where loss of neuronal stem cells, decreased proliferation of neuronal precursor cells (NPCs), and neuronal loss is observed [6]. In contrast, mouse embryonic stem (ES) cells are refractory to MCMV infection. Whether mouse neurons are refractory to infection is controversial [2]. The inconsistent results may reflect difficulties in obtaining pure neuronal cells devoid of glial cells, which are known to be permissive to MCMV [2]. Thus, damage to neurons observed in mixed cultures in some studies may reflect events secondary to glial infection and death [2].

To understand human-specific effects of HCMV, two groups have utilized neurospheres cultured from human fetal forebrain tissues [7], [8], [9]. These studies show consistently that NPCs derived from neurospheres were permissive for HCMV infection. Luo *et al.* also suggested that HCMV infection could change the neural fate specification of NPCs, biasing their differentiation toward a non-neuronal lineage [8]. These investigators also suggested that mature neurons derived from the NPCs are permissive to HCMV infection. Odeberg *et al.* reported that HCMV inhibits neuronal differentiation and induces apoptosis in human neural precursor cells [7]. In contrast to Luo *et al*., a permissive effect on neurons was not reported. Like the murine studies, the permissiveness of neurons for CMV infection is controversial in human studies.

We report an induced pluripotent stem (iPS) cell-based cellular model to investigate the effect of HCMV. NPCs can be generated from iPS cells and expanded as monolayer cultures or as neurospheres. They can also be induced to differentiate into neuronal or glial lineages [10], [11]. Thus, it is feasible to culture relatively pure cell lineages and evaluate the primary effects of HCMV and the permissiveness of different cell types, including neurons. Further, the rate of differentiation can be controlled, providing a unique perspective to study infection in relation to neuronal development.

## Materials and Methods

### Cell culture

Skin biopsy samples were collected from the shoulder of two control individuals via 4 mm full thickness punch biopsies under local anesthesia (Identification numbers V07-3 and Y1). Control subjects donated biopsies through their participation in the study “Family-Based Genome-Wide Methylation Scan in Neurocognition and Schizophrenia,” in which human subjects were enrolled at the University of Pittsburgh (PITT). The PITT Institutional Review Board approved the study, and subjects enrolled signed written informed consent prior to participation in any study procedures. The biopsy samples were digested with collagenase and primary culture initiated in T25 flasks. The cells were passaged three times before being harvested for iPS cell line generation using a standardized procedure [12]. V07-3 iPS cell line was analyzed for expression of pluripotency markers by immunostaining (Figure S1). Characterization of pluripotency markers in Y1 iPS cell line has been described elsewhere [13]. Both cell lines were adapted to mTeSR1 medium on matrigel-coated plates (Stem Cells Technology). iPS colonies were dissected manually every 6–8 days. Cell line V07-3 was used for all experiments; key results were confirmed using Y1 (data not shown). Participant V07-3 did not have elevated levels of HCMV antibodies in the serum. The methods for differentiation of iPS cells into neural stem cells, NPCs, and neurons are provided in Supporting Information S1 (Figures S3, S4, S5, and S6).

### Generation and analysis of iPS-derived teratomas

Cell suspensions were introduced into the testes of immune deficient SCID mice by modified efferent duct injection [14]. The injection pipette was advanced along the efferent duct and through the rete testes into the interstitial space where cells were injected using an Eppendorf Femtojet pressure injector. Recipient mice were routinely evaluated for palpable tumors. H&E stained sections of tumors were analyzed.

Teratoma injections were performed by the Transgenic and Molecular Core of Magee-Womens Research Institute. Animal experiments were approved by the Institutional Animal Care and Use Committee of Magee-Womens Research Institute and the University of Pittsburgh.

### Virus infection

#### AD169 strain

iPS cell colonies were dissociated manually into clumps of approximately 30–50 µm and transferred to 6-well plate in mTeSR1 medium and allowed to settle overnight. iPS cells were then infected with HCMV strain AD169 (obtained from the supernatant culture medium of infected human fibroblasts) at a multiplicity of infection (MOI) of 3 for 2 hours. Neural rosettes were dissected and transferred to matrigel-coated 24-well plates (20–30 neural rosettes/well) with NP expansion medium (see Supporting Information S1). After 3 hours, the NP expansion medium was exchanged for neurobasal medium and neural rosettes infected as described above. Separately, monolayer cultures of NPCs were grown to a confluence of approximately 80% and infected as described above. For neuronal infection, neuron-enriched cultures were infected as described above after differentiation for four weeks in neurobasal medium.

Mock infection consisted of addition of an equal volume of supernatant from non-infected fibroblasts. We also tested the effect of heat inactivated HCMV (58°C for 20 min) on human fibroblasts. No CPE was observed with either protocol until day 15 post infection (p.i.) (Figure S7a). We selected the supernatant from non-infected cells for mock infection in all the other experiments.

#### UL32-EGFP-HCMV-TB40 strain

iPS-derived neuron-enriched cultures were also infected with the recombinant UL32-EGFP-HCMV-TB40 strain as described above. This strain expresses enhanced green fluorescent protein (EGFP) fused to the C terminus of the capsid-associated tegument protein pUL32 (pp150) [15].

### Immunocytochemistry and Microscopy

Immunocytochemistry was performed as previously described [16]. Primary antibodies used were mouse anti-ß-tubulin III monoclonal antibody (clone Tuj-1, R&D System, 1∶50 dilution), mouse monoclonal anti-MAP2 (Millipore, dilution 1∶200), mouse monoclonal anti-human nestin antibody (R&D Systems, 1∶1000 dilution), mouse monoclonal anti-human/mouse SOX2 antibody (R&D Systems, 1∶200 dilution), mouse monoclonal anti-human GFAP antibody (R&D Systems, 1∶200 dilution), mouse anti-CMV immediate early antigen monoclonal antibody (Chemicon International, dilution 1∶200), mouse anti-cytomegalovirus (clone Blend; reacts with immediate early, early, and late CMV antigen preparations) monoclonal antibody (Millipore, dilution 1∶200), and rabbit polyclonal CMV-PP65 antibody (Abbiotech). Fluorescently labeled secondary antibodies were used for detection. Counterstaining was done with Hoechst 33342. Images were acquired using a Leica IL MD LED inverted fluorescence microscope.

### Fluorescence measurements of intracellular calcium concentration

To assess the neuronal function, intracellular calcium levels in infected and mock infected neuron-enriched cultures were estimated both under basal conditions and following 10 µM glutamate administration as previously described [17].

### NPCs viability

Viability of infected NPCs was assessed by flow cytometry using ‘fixable’ viability dyes (eBioscience) according to the manufacturer's recommendations. Briefly, infected cells collected on different days were washed with phosphate buffered saline (PBS) and then incubated for 30 min at 4°C with the viability dye. Following extensive washes with PBS, cells were resuspended in Fix/Perm buffer (eBioscience) to permeabilize cells, and then stained with anti-nestin antibody to confirm cellular identity during analysis. Stained cells were analyzed using a LSR-Fortessa cell analyzer (Becton, MD).

### Western blots

Cell lysates were prepared with 10 volumes of RIPA buffer (25 mM Tris–HCl pH 7.6,150 mM NaCl, 1% NP-40, 1% sodium deoxycholate, and 0.1% SDS), denatured by heating at 95°C and then separated by electrophoresis on 4–12% gradient SDS-poly-acrylamide gels, transferred onto Immobilon membranes (Milli- pore), and probed with monoclonal anti-NMDAR1 (Abcam, ab68144) and monoclonal anti Cleaved Caspase 3 (Cell Signaling, 9662). The blots were then probed with fluorescently labeled secondary antibodies (Li-Cor antibodies) to detect immunoreactivity and quantify the fluorescent signal intensities using an Odyssey Imaging scanner (Li-Cor).

### Oligonucleotide microarray experiments

Adherent monolayer culture of NPCs were either infected with HCMV Ad169 in triplicate or mock infected, with each individual sample harvested separately to provide biological replicates for expression analysis. Infected and mock infected cells were harvested 24 h p.i. Microarray experiments were performed at W.M. Keck Foundation Biotechnology Resource Laboratory (Yale University). RNA was extracted using RNeasy kits (Qiagen) and quantified by optical density (OD) measurement using a Nanodrop 1000 machine at 260 nm. An Agilent 2100 Bioanalyzer was used to estimate the RNA integrity Number (RIN). The RIN score was calculated by determining the area of the 18 s and 28 s ribosomal peaks as well as the total signal of the whole trace. Labeling was performed using the Ambion Illumina TotalPrep RNA Amplification Kit. Briefly, double stranded cDNA synthesis was performed using an oligo(dT)24 primer containing a T7 RNA polymerase promoter site. The cDNA was used as a template to generate biotin-labeled cRNA that was used for hybridization. After purification, aliquots of each sample were hybridized to Illumina platform HumanHT-12 v4 Expression. After hybridization, each array was sequentially washed, stained with CY3, and scanned on the Illumina HiScan. Arrays were individually visually inspected for hybridization defects and quality control procedures were applied, as recommended by the manufacturer of the arrays. For array readout, we used Illumina Feature Extraction Software (Genome Studio 11.0). Hybridization controls were evaluated to ensure the experiment had no errors. Data files were normalized using quantile normalization method. Differentially expressed genes were identified using the Student *t* test and significance was defined as *P*<0.05 (uncorrected for multiple comparisons), with fold change (FC) of FC<0.8 or FC>1.2. The gene expression microarray data on human neuronal progenitor cells (NPCs) been deposited in Gene Expression Omnibus (GSE35295).

### Library Construction And High Throughput Sequencing

Strand specific sequencing libraries were constructed by modifying the Truseq RNA Sample Prep Kit (Illumina, CA) protocol. Our strand specific protocol is based on the published method of Levin et al. [18]. Briefly, 2 µg of total RNA was subjected to poly-A purification, fragmentation and first strand cDNA synthesis following the protocol recommended in the TruSeq RNA Sample Prep Kit. For second strand cDNA synthesis, we added 2 µl 10× Superscript RT buffer (LifeTech), 8 µl 10× NEBNext second strand synthesis (dNTP-free) reaction buffer, 2.5 µl of 10 µM nucleotide solution containing dUTP instead of dTTP, 4 µl of NEBNext second strand enzyme mix to the 20 µl first strand cDNA, brought the reaction to a volume of 100 µl with water and incubated at 16°C for 2.5 hours. The resulting double stranded cDNA was purified using the Ampure beads and end repair, adenylation and adapter ligation was performed as described in the Truseq RNA protocol with the exception that only 1 µl of adapter was used for ligation. Following adapter ligation and purification, the resulting cDNA was digested with USER enzyme in a 50 µl reaction containing 5 µl of 10× PCR reaction buffer (Qiagen), 1 unit of USER enzyme (NEB). The reaction was incubated for 15 minutes at 37°C and the USER enzyme was inactivated by heating to 95°C for 5 minutes. The resulting library was purified using Ampure beads prior to PCR amplification. PCR and PCR clean up was performed as described in the TruSeq protocol.

One hundred base single read sequencing was performed on the libraries using a HiSeq 2000 sequencer (Illumina). Roughly 37.5 gigabases of sequence was produced for each sample.

### Sequencing Analysis For Viral Read Counts

In order to prevent erroneous mapping of human sequences onto the viral genome, reads that mapped to the human genome were removed from the sequencing files prior to alignment with the viral genome. A sliding window approach was used to align a forty base pair subsequence from the reads to the human genome using the bowtie short read aligner [19]. During each iteration, reads containing a 40 bp subsequence mapping to the human genome was removed from the analysis. Then the subsequence used for alignment was offset by five bases and the reads that had not aligned previously were mapped back onto the genome. This cycle was repeated until the sliding 40 bp window reached the end of the read. The reads that survived this subtraction procedure were mapped onto the viral genome using bowtie and viral read counts were calculated based on this mapping.

## Results

**Figure 1:**
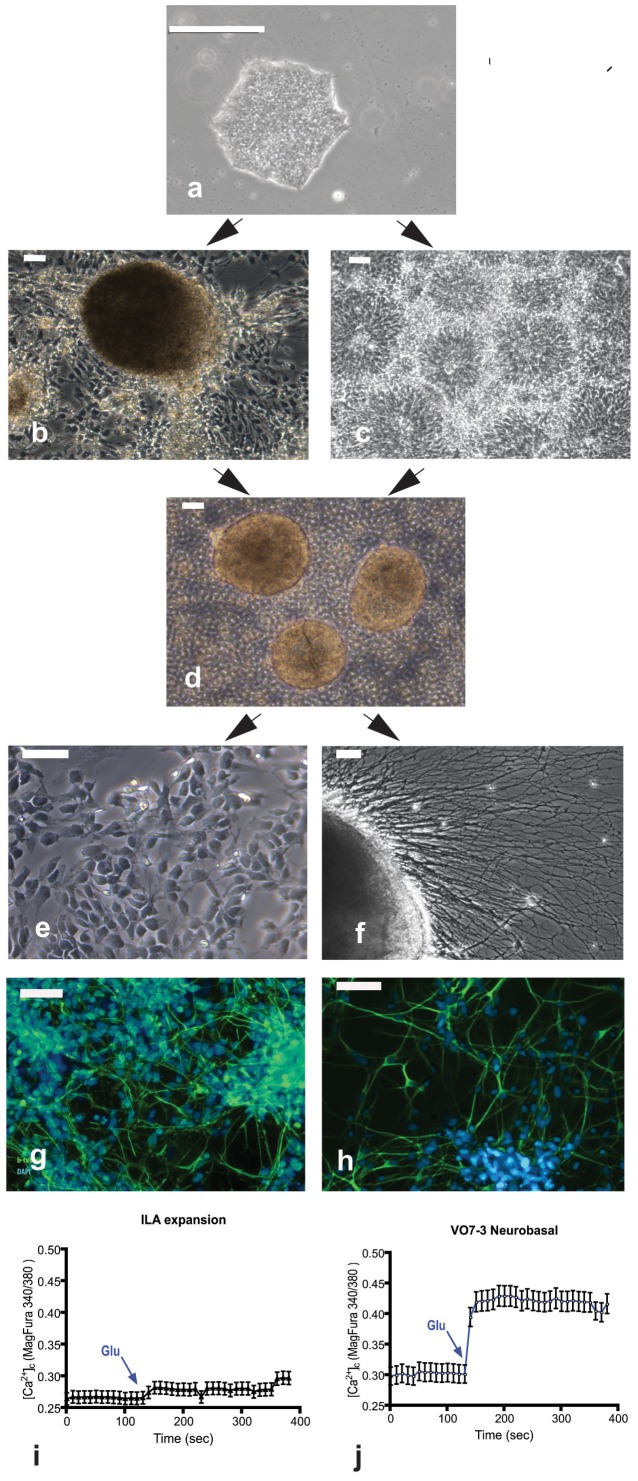
Neuronal differentiation from iPSCs generated from fibroblasts. (a) Typical morphology of an iPS colony cultured for 7 days on matrigel with mTeSR1 medium. (b) Spherical cluster of cells containing neural stem/progenitor cells. (c) neural rosettes. (d) Neurosphere-like structures formed 1 day after culturing in suspension dissected spherical cluster and neural rosettes. (e) neural progenitor cells (NPCs). (f) neuron differentiating from neurosphere-like structures. (g–h) Staining of neurons with ß-tubulin III (Tuj1) (g) and MAP2 (h). Glutamate administration (10 µM) in NPCs cultures (i) did not evoked a significant calcium influx (Ca^2+^ basal levels: 0.26 ± 0.02; after glutamate: 0.27 ± 0.03; n = 128; not significant), whereas higher calcium basal levels were recorded in NPCs-neurons (j) (0.30 ± 0.02; n = 136; p<0.005 compared to NPCs calcium basal levels) and glutamate administration evoked a quicker and stronger response in term of Calcium influx (0.42 ± 0.03; n = 136; p<0.005 compared to calcium basal levels). The data are representative of four different experiments. The significant increase of glutamate-mediated Ca2+ influx suggests that the iPS-derived neurons are functional. Scale bar is 50 µm.

Standard characterization of the iPS cells was initially conducted. No abnormalities were detected following karyotyping, and the clones stained as expected with NANOG, Oct4, TRA60, TRA811, OSX2 and SSEA4 (Figure S1). Following injection into the testes of SCID mice, palpable tumors developed between two and four months after injection. H&E stained sections of tumors are shown (Figure S2). Differentiation of human iPS cells sequentially produces distinctive structures composed of neural lineages identifiable with cell specific markers. The stages of differentiation include neural rosettes (composed of NSCs), NPCs, and neurons (Figure 1). Incubation with glutamate (10 µM) significantly increased Ca^++^ influx into the neurons, but not NPCs. Each of these lineages was infected with HCMV.

### Human iPS cells are not permissive to HCMV infection

**Figure 2:**
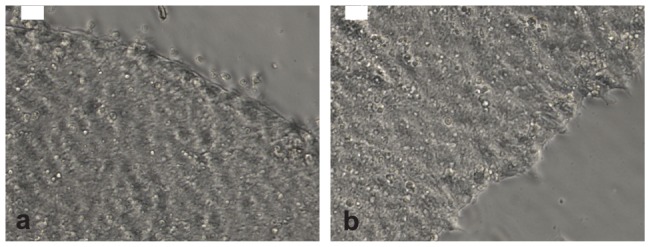
Microphotographs of HCMV-infected (a) and mock infected iPS cells. Scale bar is 50 µm.

To test whether human iPS cells are refractory to HCMV infection as observed for mouse ES cells [20], iPS cells were infected with HCMV at an MOI of 3 and cultured for up to 15 days. No significant morphological changes were observed in HCMV infected iPS cells (Figure 2a) compared with mock infected cells (Figure 2b). Furthermore, expression of immediate early, early and late HCMV antigens were not detected by immunostaining (data not shown), indicating that human iPS cells are not permissive for HCMV infection.

### HCMV infection of NSCs impairs neuronal differentiation

**Figure 3:**
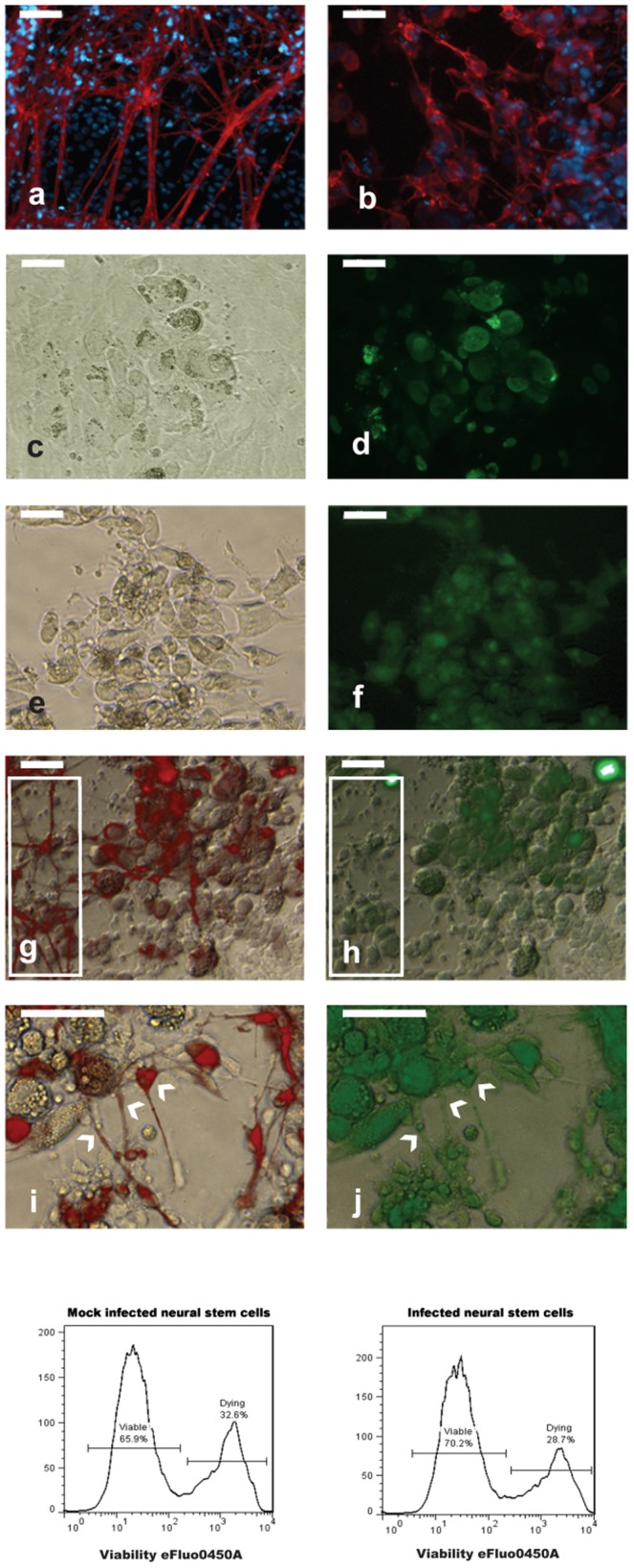
Effect of HCMV on differentiation of neural rosettes. *Top panel*. Identification of neurons differentiating in mock infected (a) and infected (b) cultures by Tuj 1 immnostaining (red). The presence of HCMV antigens in infected cells displaying CPE (c, e) was determined by staining for pp65 (d) and blend (f, see material and methods). (g–j): Analysis of neurons differentiating from infected neural rosettes. Co-immunostaining for Tuj1 (red) and pp65 (green) showed that cells expressing the virus antigen (h) strongly stain for Tuj1 (g). Most of the cells with neuronal morphology stain for Tuj1 (g, within the box, left) but not for pp65 (g, within the box, left). Co-expression of ß-tubulin III (i) and pp65 (j) was observed only in a small fraction of neurons (indicated by arrowheads). Scale bar is 50 µm. *Bottom panel*. FACS analysis of neural stem cells viability after HCMV infection (day 15 p.i.).

To investigate whether HCMV alters the differentiation of neural stem cells as reported with MCMV infection [6], neural rosettes (a distinct class of neural stem cells with a broad differentiation potential, see Figure 1c) were isolated manually and plated in 24-well plates at a density of 20–30 rosettes/well. The characteristic cellular organization of neural rosettes (wedge-shaped cells arranged radially around a central cavity) allows for their easy isolation from the differentiating iPS cells. After 3 hours to allow for attachment to the surface of the plastic plate, the neural rosettes were infected at MOI of 3 with HCMV and cultured in neurobasal medium. We initially monitored the morphological changes to assess the development of cytopathic effects (CPE) and neural differentiation. At day 3 post infection (p.i.) neuron-like cells appeared in the cultures and their number increased with time. Most of the neurons differentiated in infected cultures showed abnormal morphology compared to neurons differentiated in mock infected cultures (Figure 3a–3b). A moderate CPE (increased cell volume as well as the presence of intranuclear inclusion bodies) was observed in the infected cultures (Figure 3c, 3e). Immunostaining for pp65, a major component of the viral tegument, showed the presence of the protein in a substantial majority of the cells displaying CPE (see Figures 3d). The presence of viral antigens was also assayed by staining for immediate early, early and late HCMV antigens (Figure 3f). Immunostaining with ß-tubulin III (clone TuJ1) showed a substantial difference in neuronal morphology between infected and mock infected cultures, suggesting that HCMV affects neural differentiation (Figure 3a–b). Furthermore, immunocytochemical analysis revealed no staining for pp65 in most of the neurons differentiated from neural stem cells (see boxed areas, Figure 3g–h). Weak to moderate staining of pp65 was detected in only a few neurons (Figure 3i–3j). It is noteworthy that a conspicuous fraction of infected cells (expressing the viral antigen pp65) displayed a strong staining for Tuj1 (Figure 3g–h), whilst in mock infected cultures only cells with neuronal morphology were stained with Tuj1 (Figure 3a). Taken together, these results suggest that HCMV impairs neural differentiation in neuronal stem cells.

At day 15 p. i. approximately 70% of cells were viable regardless of the infection status (Figure 3, bottom panel). To evaluate whether HCMV can replicate following infection of neural rosettes and their progeny, the supernatant from infected and mock infected cells was collected at day 15 and used to infect human fibroblasts. At day 7 p.i., CPE was observed only in culture exposed to the medium collected from infected cultures (Figure S7c–d).

### Susceptibility of NPCs to HCMV infection

**Figure 4:**
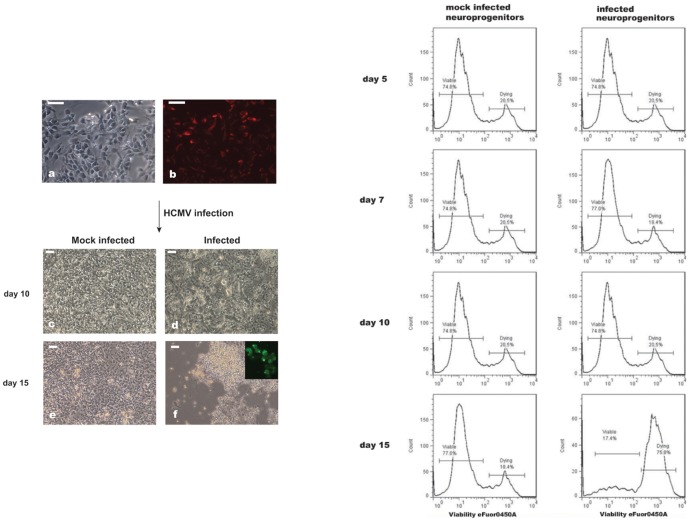
HCMV infection of NPCs. *Left panel*. (a) microphotograph of NPCs before infection. The percentage of progenitors in the culture approach 90% as showed by staining with nestin (b). Cytopathic effect developed in the infected cultures (d, f). Microphotographs of mock infected NPCs are depicted in (c) and (e). Scale bar is 50 µm. *Right panel*. FACS analysis of NPCs viability after HCMV infection gated on the nestin-positive cells.

Neurosphere-like structures (Figure 1d) were dissociated in single cells and expanded as monolayer cultures of NPCs (Figure 4a). Immunocytochemical analysis showed that at the beginning of the experiment, the majority of the cells were nestin positive (Figure 4b). Cells were infected with HCMV at MOI of 3 and morphological changes and development of CPE were monitored (Figure 4c–f). The viability of HCMV infected NPCs was analyzed by assessment of extracellular membrane permeabilization, quantified by flow cytometry (Figure 4, right panel).

Development of CPE was first observed at day 10 p. i. (Figure 4d). Figure S8e, h shows immunostaining of the cells displaying CPE for CMV early immediate and nestin antigens, respectively. Until day 10, FACS analysis showed no apparent difference with regard to cell viability between the infected and mock infected cells (Figure 4, right panel). Cell death became more obvious at day 12 p.i. (data not shown) and at day 15 p.i. the percentage of dead cells rose to approximately 76% (Figure 4, right panel). These results suggest that human iPS-derived NPCs are permissive for HCMV infection. The medium from infected and mock infected NPCs was collected at day 15 p.i. and used to infect fibroblasts. Fibroblasts exposed to the medium collected from infected cultures exhibited a CPE, showing that NPCs can support HCMV replication (Figure S7e–f).

RNA-seq was performed on NPCs infected with HCMV for 24 hours. The RNA-seq expression analysis showed expression of HCMV genes UL36–38 and UL84 required for the viral DNA replication (Table S1) [21].

### HCMV infection affects the expression of neuronal-related genes

Considering the effect of HCMV infection on neural differentiation, we reasoned that the virus may affect the regulation of the neural related genes during the early stage of infection. To test this hypothesis, we infected monolayer cultures of NPC at MOI of 3. NPCs were harvested after 24 hours for mRNA analysis. Gene expression data sets for infected NPCs were compared to those of mock infected NPCs harvested at the same time. We found that during the first 24 hours p.i., the viral infection changed the expression of 55 genes; 30 were up-regulated and 25 were down-regulated (p<0.05, uncorrected, fold changes less than 0.8 or greater than 1.2, see Table 1). 14 out of 55 dysregulated genes were related to neuronal metabolism or neuronal differentiation (TH transcript variant 2, TH transcript variant 3, PNMA3, GAD1, BHLHE22, KCNQ2, GRIN1, septin 4, NRXN2, BEX5, OTP, SEMA3A, ABLIM1, PRNP, and APP) (Table 1). Pathway analysis using Ingenuity software showed that up-regulated genes were involved mostly in psychological disorder, neurological diseases, and infectious diseases (Table 1).

**Table 1:** Significant gene expression changes following HCMV infection of neural progenitor cells.

Gene Name	FC	t-test p	Developmental Disorder	Infectious Disease	Neurological Disease	Psychological Disorders
LOC100008589	1.423	0.0008				
**PNMA3**	1.2729	0.0021				
**TH**	1.2788	0.0047			X	X
**GAD1**	1.4017	0.0028			X	X
HOPX	1.4859	0.0075				
**BHLHE22**	1.2528	0.0148				
HMP19	1.2565	0.0355			X	
AKR1C2	1.2661	0.0213			X	
SNAP91	1.2747	0.0497			X	X
**KCNQ2**	1.2001	0.0031			X	X
PPFIA4	1.2008	0.0079				
**GRIN1**	1.2009	0.0249	X	X	X	X
NGLY1	1.201	0.0202				
LOC389816	1.2024	0.0473				
ZFAND2A	1.2111	0.01				
RAP1GAP	1.2113	0.0104			X	X
LOC678655	1.2121	0.0104				
LOC391833	1.2132	0.0158				
CCNA1	1.2132	0.0208				
LEMD1	1.2146	0.0079				
ZNF397	1.2183	0.0195				
AKR1C4	1.2189	0.0446				
**septin 4**	1.2194	0.0072				
WSB2	1.2194	0.0063				
**NRXN2**	1.2208	0.0393				
**BEX5**	1.2215	0.0051			X	
**OTP**	1.2254	0.0443				
TCEAL2	1.2293	0.0272				
TSPO	1.2323	0.0219			X	X
C3ORF26	1.244	0.0073				
**SEMA3A**	0.7175	0.0044			X	X
S1PR3	0.7221	0.0076			X	
CXCL6	0.7247	0.0024				
**ABLIM1**	0.7745	0.0053			X	X
SDC2	0.7892	0.0024			X	X
FSTL1	0.7943	0.0071				
BSG	0.7952	0.0082	X			
MGP	0.5095	0.0422			X	
COL8A1	0.6147	0.0264			X	X
FRZB	0.6263	0.0202			X	
COLEC12	0.6647	0.0162			X	
CYP26A1	0.7165	0.0327				
ITGA11	0.7178	0.0303			X	X
MCM4	0.7518	0.0175				
GPR177	0.7519	0.0251				
**PRNP**	0.7677	0.01	X	X	X	X
EN2	0.771	0.0211				
LOC402251	0.7765	0.026				
PABPC4	0.7799	0.0355				
**APP**	0.7863	0.0105	X	X	X	X
RRM2	0.7896	0.0299			X	
EMID2	0.7936	0.0149				
MCM5	0.7938	0.0227			X	X
MCM2	0.7946	0.0136				
PRPF4B	0.7762	0.0004			X	X

List of genes significantly dysregulated in HCMV-infected neural progenitor cells 24 hours p.i. (p<0.05). FC: fold change; t-test p: p value for infected vs mock infected t-test. ‘X’s in squares indicate genes implicated in indicated categories of disorders at p<0.05. Neuronal-related genes are in bold. Ingenuity Pathways Analysis (IPA) results report p value ranges: Psychological disorders 1.62E-06-4.28E-02. Neurological Disease 1.26E-05-4.8E-02; Developmental Disorder 4.35E-05-2.7E-02; Infectious Disease 4.35E-05-3.23E-02.

### Mature neurons degenerate following HCMV infection

**Figure 5:**
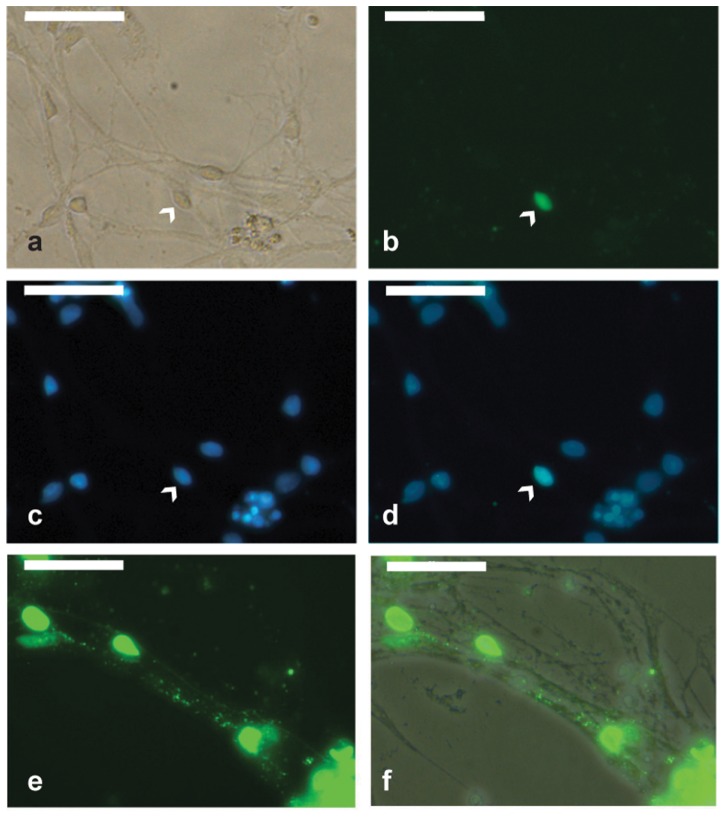
Mature neurons exposed to HCMV. Neuron-enriched cultures were infected with HCMV an MOI of 3. Expression of HCMV proteins was detected only in a small fraction of neurons (indicated by arrowhead) at day 3 post infection. (a) Bright-field. (b) Staining for early immediate, immediate and late HCMV antigens. (c) Nuclei counterstained with Hoechst. (d) Overlay of HCMV antigens and bright-field. (e–f) Staining for early immediate, immediate, and late HCMV antigens shows the presence of vial proteins (green) along neural processes at day 5 post infection. (f) Overlay of viral antigens and bright-field. Scale bar is 50 µm.

Neuron-enriched cultures were prepared by exploiting the differential ability of neurons and glial cells to adhere to fibronectin-coated plates (see materials and methods). This strategy enabled us to harvest cultures containing 80–90% of ß-tubulin III positive cells; i.e., neurons (Figure S9). After 4 weeks of culture in neurobasal medium, neuron-enriched cultures were infected with HCMV at an MOI of 3. Infected neurons were monitored for morphological changes and assayed for localization of viral proteins by staining them with a mixture of monoclonal antibodies that react with immediate early, early, and late HCMV antigens. At day 3 p.i., the expression of HCMV proteins was observed in the soma of rare neurons (Figure 5a–d). Their frequency did not increase substantially at day 5, when expression of viral antigens was also detected along neural processes (Figure 5e–f).

**Figure 6:**
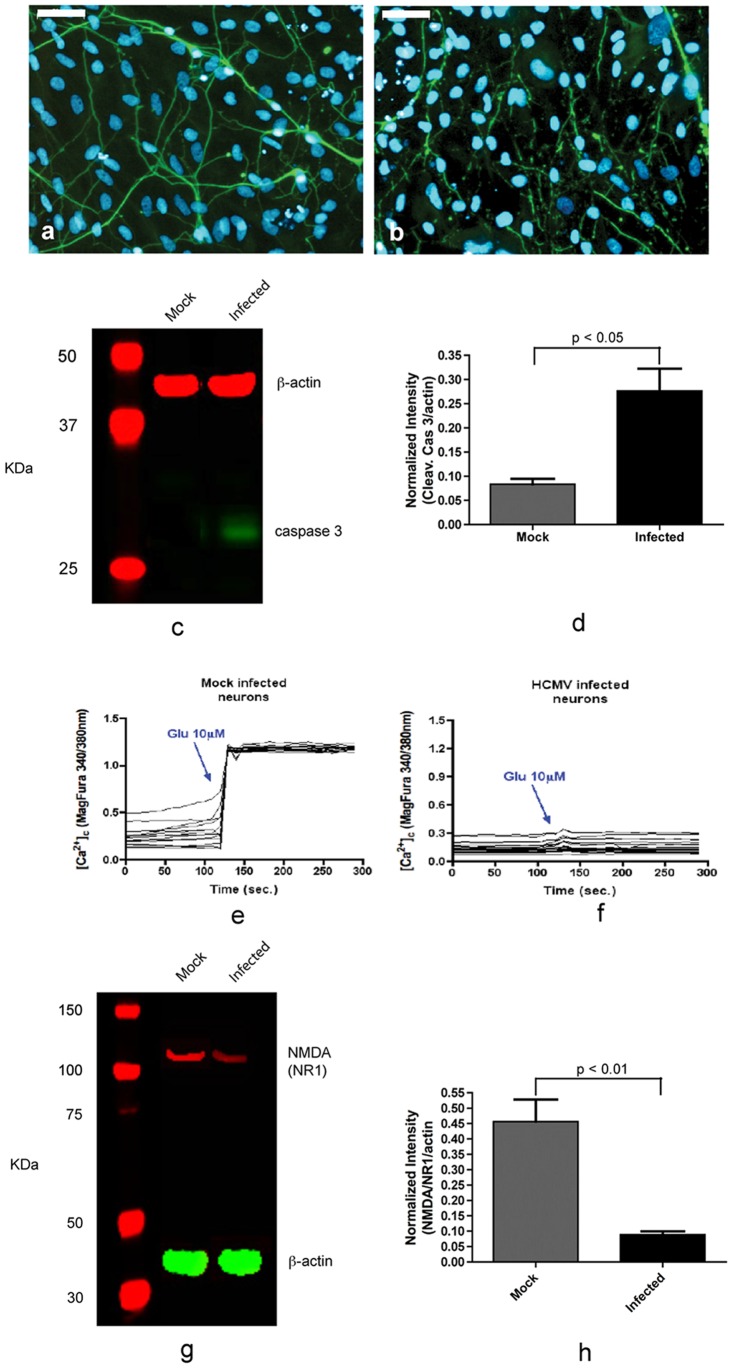
Effect of HCMV on neural viability and function. HCMV reduces neuronal viability and down-regulates NR1 subunit of NMDA receptor. At day 7 post infection most neurons showed degeneration as highlighted by immunostaining for Tuj1 (b), whilst mock infected neurons maintain their integrity (a). (c) Immunoblot showing activation of caspase 3 in infected neuron cultures. (d) Normalized cleaved caspase 3 expression from data in (c). (e–f): Measurements of calcium influx in mock infected (e) and infected (f) neurons induced by administration of 10 µM glutamate [Glu]. (g) Immunoblot showing reduced level of NR1 subunit in HCMV infected neuron cultures. (h) Normalized NR1 expression from data in (g). Scale bar is 50 µm. The data represent an average of three independent experiments.

Between days 5–7 we observed an overall reduction of neuronal number in infected neuron-enriched cultures when compared with mock infected neuron-enriched cultures. Moreover, we observed that most of the neuronal processes were loosely attached to the surface of the plate (data not shown). Immunoreactivity for Tuj1 at day 7 p.i. appeared punctated on most of the neurons in the infected cultures, indicating neural degeneration (Figure 6b). Mock infected cells remained viable (Figure 6a). Taken together, these results suggest that HCMV replication is inhibited in most of the infected neurons, but the presence of HCMV in the cells may induce apoptosis. To test this possibility, mock infected and infected neurons were investigated for the cleaved caspase 3. Western blotting analysis carried out in neurons at day 3 p.i. showed that the level of cleaved caspase 3 was 3-fold higher in neurons of infected neuronal cultures. Thus, neural degeneration observed in infected neuronal culture may be caused by apoptosis (Figure 6c–d).

### Effects of UL32-EGFP-HCMV-TB40 strain

To investigate whether the effects observed with HCMV strain AD169 were also noted with another HCMV strain, we used the recombinant UL32-EGFP-HCMV-TB40 strain. This strain expresses enhanced green fluorescent protein (EGFP) fused to the C terminus of the capsid-associated tegument protein pUL32 (pp150) [15]. Starting from day 6 p.i., EGFP expression was observed in non-neuronal cells but not in neuronal somas. Most of the EGFP-expressing cells exhibited a cytopathic effect (CPE, Figure S10a–d)). Infected neuronal cultures were monitored for 12 days. During this period of time, no EGFP-expressing neurons were observed (Figure S10e–f). Immunostaining of infected cultures with ß-tubulin III (clone TuJ1) confirmed a consistent lack of EGFP expression in neuronal cells (Figure S11). These results suggest that refractoriness of neurons for HCMV is not restricted to the attenuated HCMV strain AD169 but also shared by the clinical isolate TB40.

### Functional analysis of HCMV infected neurons

We further investigated whether HCMV infection causes functional changes in neurons, by estimating the calcium influx induced by administration of 10 µM glutamate. Neurons differentiated for four weeks in neurobasal medium were infected at MOI of 3. At day-3 p.i., the neurons showed lower intracellular calcium basal level compared to mock infected cells (data not shown). Furthermore, when assayed for glutamate-induced calcium influx, significant evoked intracellular calcium increase was not detected in HCMV-infected neurons after glutamate administration (Figure 6f), in contrast to mock infected neurons (Figure 6e). To investigate whether the absence of elevation of intracellular calcium level is caused by an alteration in the levels of N-methyld-D-aspartate receptor (NMDAr) in the infected neurons, western blots of the NR1 regulatory subunit of NMDAr were obtained. Western blotting analysis showed that the level of NR1 in neurons of infected cultures was approximately 4-fold lower (Figure 6g–h).

## Discussion

We have investigated the effects of HCMV infection on several neural cell lineages derived from iPS cells. We observed that iPS cells and iPS-derived neurons are not permissive to HCMV infection. In contrast, NPCs are fully permissive, suggesting a stage in neural development that is particularly vulnerable to HCMV infection. Our results are largely consistent with studies in murine cells [20] and neurospheres from human fetal forebrain tissues [7], [9].

We also employed iPS-derived neural rosettes [22] to evaluate the impact of HCMV on neural differentiation. Cells displaying CPE also showed strong imunoreactivity for ß-tubulin III (Tuj1), suggesting that the infected cells are committed to a neural fate, but neuronal differentiation is inhibited. Thus, in HCMV infected cultures of neural stem cells, the number of ß-tubulin III positive cells does not necessarily reflect the number of neurons. Hence we could not determine the percentage of the neurons differentiated in infected cells *versus* mock infected cultures.

Excitatory signaling has been shown to play a critical role in the induction of neural differentiation in different brain areas through changes of cellular polarization and calcium homeostasis [23], [24], [25], [26]. In the present study, global gene expression analysis of NPCs infected for 24 hours showed that HCMV causes dysregulation of genes involved in the modulation of cellular excitability (Table 1). It is conceivable that HCMV-mediated dysregulation of genes encoding enzymes and ion channels involved in inhibitory modulation of neuronal activity may cause the impairment of neuronal differentiation observed in infected NPCs. In particular, we found a significant up-regulation of Tyrosine Hydroxylase (TH), Glutamate decarboxylase (GAD1), and KCNQ2 (encoding Kv.7 potassium channel, involved in the control of the resting potential and neuronal differentiation) [27]. Furthermore, SEMA3A, a crucial protein for the normal neuronal pattern development [28], [29] was significantly down-regulated in infected NPCs. This observation suggests that HCMV infection impairs the control of the axons and dendrites outgrowth in infected NPCs.

Challacombe *et al.* [30] reported that human cytomegalovirus (HCMV)-infected cells showed differential expression of two clusters of coexpressed human genes: one containing some genes identified previously [8], and another that was largely unique to their analysis. The list included genes involved in transcriptional regulation, oncogenesis, and cell cycle regulation, which were more prevalent in cluster 1, and genes involved in immune system regulation, signal transduction, and cell adhesion, which were more prevalent in cluster 2. They stressed the advantages of using different methods to analyze gene expression data. Our results are not comparable to the previous studies because they used different cell types and and different HCMV strains.

NPCs showed increased susceptibility to the HCMV infection in comparison with NSCs. Fifteen days after HCMV infection, approximately 17% of NPCs survived (Figure 4, right panel), with most dead cells floating in the medium (Figure 4f), whilst the percentage of living neural stem cells was approximately 70% (Figure 3, bottom panel). Exploratory analysis showed that HCMV affects the expression of genes related to neural metabolism or neuronal differentiation (Table 1). Microarray analysis of infected NPCs demonstrates significant down-regulation of the MCM class of genes together with RRM (Table 1), which are involved in DNA replication. Shechter *et. al.* have reported that Mcm proteins are crucially required for initiation of DNA replication [31] and also inhibition of Mcm2–7 function during S phase causes a rapid termination of DNA synthesis [32]. Our results may suggest previously unrecognized link between HCMV infection and viability of NSCs and NPCs through dysregulation MCM gene class.

Immunoreactivity for viral antigens was not observed in most of the cells with neuronal morphology expressing ß-tubulin III. Co-expression of viral antigens and ß-tubulin III was observed only in a small fraction of neurons (Figure 3i–j). Furthermore, most of the neurons differentiated from infected neural stem cells showed abnormal arborization and branching (Figure 3b). Interestingly, despite inhibition of neural differentiation, ß-tubulin III accumulated in infected cells, suggesting that HCMV interferes also with mechanisms that control ß-tubulin III expression [33].

The permissiveness of neurons to HCMV remains controversial. Cheeran *et al*. [2] suggested that MCMV does not actively replicate in murine neurons, but Luo *et al.* reported that human neurons are fully permissive to HCMV [9]. In our studies, immnunostaining for immediate early, early and late genes showed nuclear localization in <1% of neurons (Figure 5a–d). Viral proteins were also detected along neural processes in some cells (Figure 5e–f). Thus, neurons derived from human iPS cells are not generally permissive to HCMV. The small subset of neurons expressing viral antigens may represent a sub-type that supports HCMV replication. Indeed, stable expression of host genes under the control of the HCMV promoter is achieved in cholinergic neurons, whilst gluatamatergic, GABAergic and noradrenergic neurons show relatively low gene expression [34]. The relative permissiveness of certain neurons may be due to expression of specific transcription factors required to activate the major immediate-early HCMV enhancer [35]. Such factors may not be expressed in all neurons.

Activation of caspase 3 in neurons in infected cultures at day 3 p.i. (Figure 6c–d) shows that HCMV induces apoptosis in neuronal cells. The infection also appears to impair neuronal function, as noted from the marked reduction in Glu-induced Ca^2+^ influx (Figure 6e–f). The reduction may be explained by the down-regulation of NMDAr in infected culture (Figure 6g–h). This result is in agreement with a previous study in murine model which reported that murine cytomegalovirus infection causes a reduction of NMDA expression in the hippocampal neurons of neonatal mice and primary neuronal cultures [3].

The earlier studies with rodent and human studies utilized neural stem cells and their progeny at different stages of differentiation. Our system enables evaluation of more homogenous cell types under controlled conditions. Further, the availability of large numbers of cells opens the potential for more sophisticated analyses, such as effects of chronic infection [8]. Combined with high throughput, unbiased RNA sequencing technologies [36], comprehensive gene expression studies of homogenous cell types is thus feasible. Our models could also be used to investigate host genetic variation in relation to HCMV infection.

There are important limitations to the present studies. Like other cellular models, it is difficult to recapitulate all aspects of neural differentiation *in vitro*. In particular, we did not evaluate the role of immunologic factors while investigating the effects of HCMV infection; further elaboration of the model is thus necessary. Specific glial cell lineages were also not analyzed as derivation of glial cells from iPS cells is challenging at present. Additional HCMV strains also need to be evaluated. Some of the analyses are descriptive; they set the stage for future quantitative studies.

In conclusion, we describe a model for investigation of HCMV effects on human neural cells following differentiation of human iPSCs. This model could also be used for other neurotropic viruses.

## Supporting Information

Figure S1 **Expression of pluripotency markers in V07-3 iPS cell line by immunocytochemistry.**
(TIF)

Figure S2 **Histologic sections of teratomas resulting from** ***in vivo*** **differentiation of human iPS V07-3 cell line.** Tissues from all three primary cell lineages (endo-, meso-, and ectoderm) were formed in individual teratomas. **a**, grastrointestinal tract of endodermal origin. **b**, muscle of mesodermal origin. **c**, neuroectoderm.
(TIF)

Figure S3 **Immunocytochemical analysis of iPS cells and their progeny in mTeSR1 medium, Neural Selection Medium and Neural Expansion Medium.** Scale bar is 50 µm.
(TIF)

Figure S4 **Immunocytochemical analysis of spherical cluster of cells generated during neural differentiation of iPS cells.** Scale bar is 50 µm.
(TIF)

Figure S5 **Immunocytochemical analysis of neurosphere-like structures.** Scale bar is 50 µm.
(TIF)

Figure S6 **Immunocytochemical analysis of iPS-derived neurons.** Scale bar is 50 µm.
(TIF)

Figure S7 **Evaluation of viral infection protocols.** (a) Heat inactivation of HCMV was tested as an alternative form of mock infection. Heat-inactivated HCMV did not produce any CPE in comparison with (b) HCMV-infected fibroblasts. Media were collected from mock infected neural rosettes, mock infected neural progenitor cells, HCMV-infected neural rosettes, and HCMV-infected neural progenitor cells at day 15 p.i., cleared by centrifugation and diluted in the medium to infect fibroblasts. CPE was observed, starting from day 5 p.i., in fibroblast cultures exposed to the supernatant collected from HCMV-infected neural rosettes (d) and HCMV-infected neural progenitor cells (f), but not from mock infected neural rosettes (c) or mock infected neural progenitor cells (e). Scale bar is 50 µm.
(TIF)

Figure S8 **Immunocytochemical analysis of infected neural progenitor cells.** Morphology of mock infected neural progenitor cells is depicted in (a). Degenerative change in infected cells is characterized by a round morphology with increased size (b, d, g), and detachment of cells from the surface of the dish (c). Expression of CMV immediate early gene (e) and nestin (h) in infected neural progenitor cells showing CPE. Cells were counterstained with Hoechst (f, i). Scale bar is 50 µm.
(TIF)

Figure S9 **Tuj1 immunostainig of neuron-enriched cultures.** (a) bright-field, (b) Tuj1, (c) Hoechst, (d) merge. Scale bar is 50 µm.
(TIF)

Figure S10 **Infection of neuron-enriched cultures with UL32-EGFP-HCMV-TB40 strain.** This recombinant HCMV strain expresses EGFP under the control of tegument protein pUL32. Microphotographs of infected neuronal cultures at day 6 (a–b), day 11 (c–d) and day 21. Scale bar is 50 µm.
(TIF)

Figure S11 **Mature neurons exposed to UL32-EGFP-HCMV-TB40 strain.** Neuron-enriched cultures were infected with UL32-EGFP-HCMV-TB40 strain an MOI of 3. Expression of EGFP under the control of the HCMV UL32 gene was not detected in neurons staining with Tuj1. Nuclei counterstained with Hoechst. Scale bar is 50 µm.
(TIF)

Supporting Information S1 (DOCX)

Table S1 **RNA-Seq analysis of HCMV transcripts in infected NPCs.**
(XLSX)
