# Correction: Human Induced Pluripotent Stem Cell-Derived Models to Investigate Human Cytomegalovirus Infection in Neural Cells

**DOI:** 10.1371/annotation/ea66e8f1-9f80-422f-8836-308a4cdc8ae4

**Published:** 2014-01-02

**Authors:** Leonardo D'Aiuto, Roberto Di Maio, Brianna Heath, Giorgio Raimondi, Jadranka Milosevic, Annie M. Watson, Mikhil Bamne, W. Tony Parks, Lei Yang, Bo Lin, Toshio Miki, Jocelyn Danielle Mich-Basso, Ravit Arav-Boger, Etienne Sibille, Sarven Sabunciyan, Robert Yolken, Vishwajit Nimgaonkar

Figure 4 is incorrect. Please view the correct Figure 4 here: 

**Figure:**
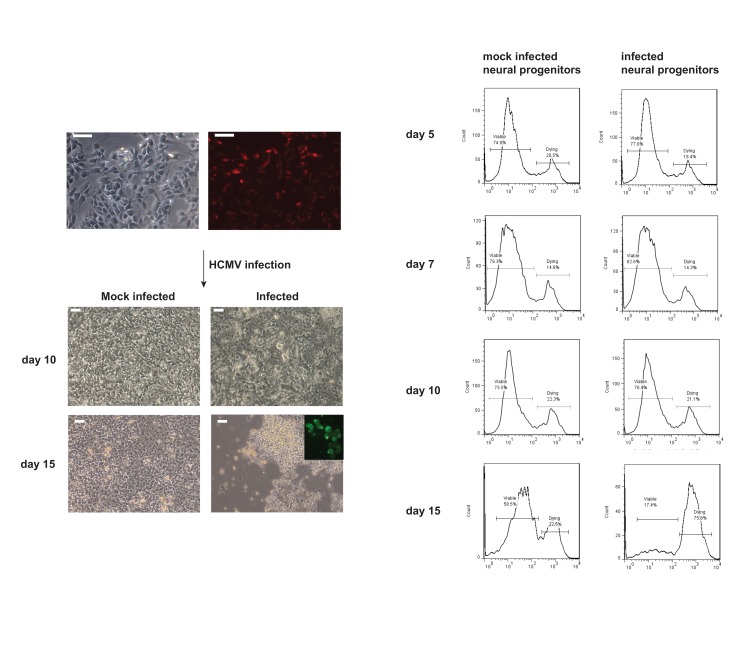


The title and legend of Figure 4 are correct. 

