# Supplementary figures and images for: Human Induced Pluripotent Stem Cell-Derived Models to Investigate Human Cytomegalovirus Infection in Neural Cells
Source: PLoS One. 2012 Nov 27;7(11):e49700. doi: 10.1371/journal.pone.0049700 (PMC3507916; doi:10.1371/journal.pone.0049700)

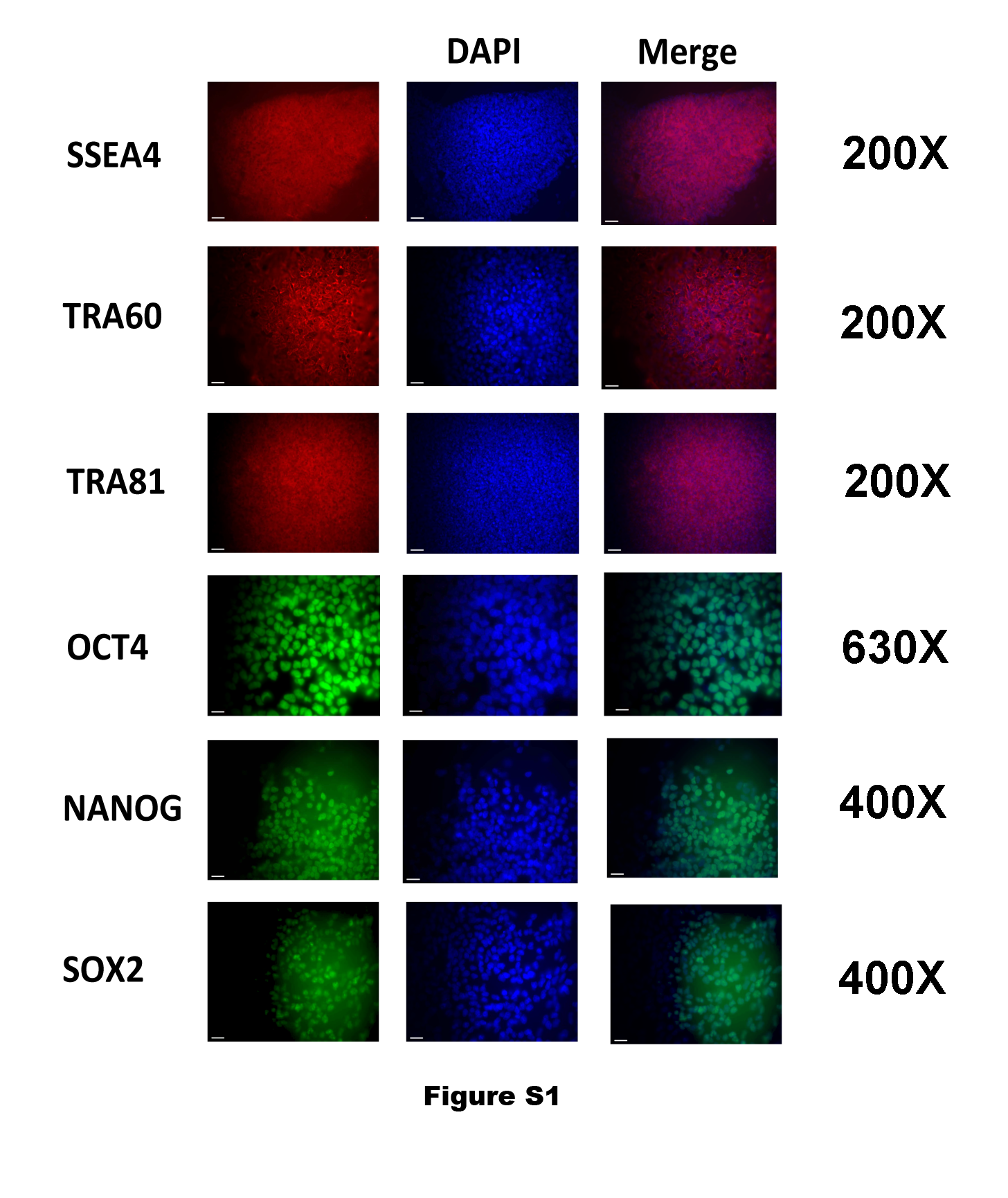

Supplement: Figure S1 — Expression of pluripotency markers in V07-3 iPS cell line by immunocytochemistry. (TIF) [file pone.0049700.s001.tif]

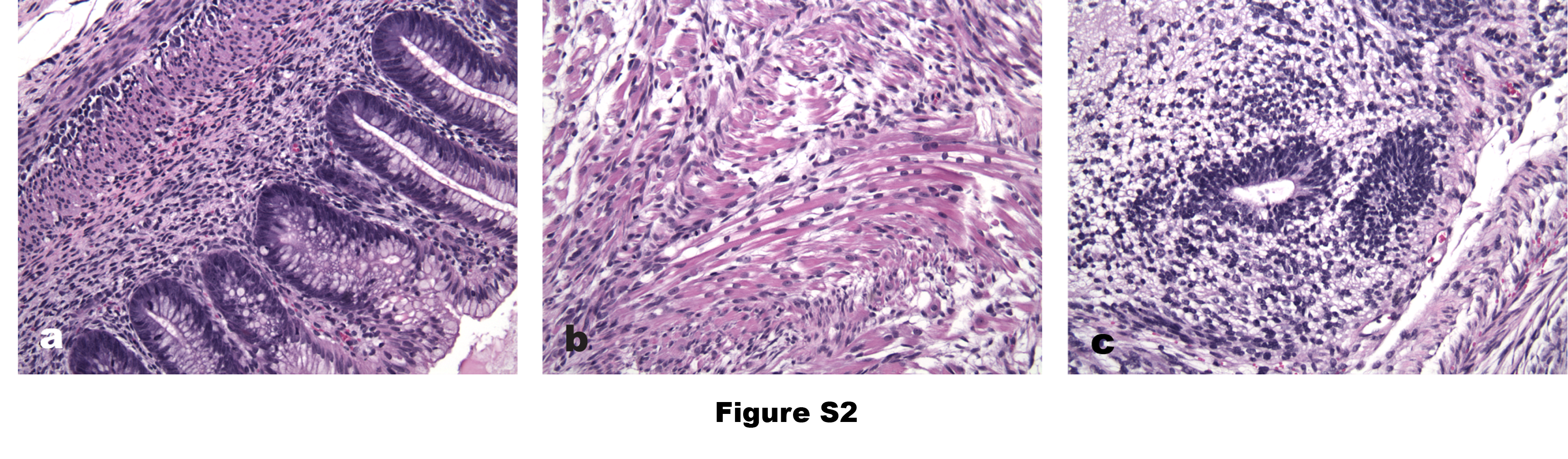

Supplement: Figure S2 — Histologic sections of teratomas resulting from in vivo differentiation of human iPS V07-3 cell line. Tissues from all three primary cell lineages (endo-, meso-, and ectoderm) were formed in individual teratomas. a, grastrointestinal tract of endodermal origin. b, muscle of mesodermal origin. c, neuroectoderm. (TIF) [file pone.0049700.s002.tif]

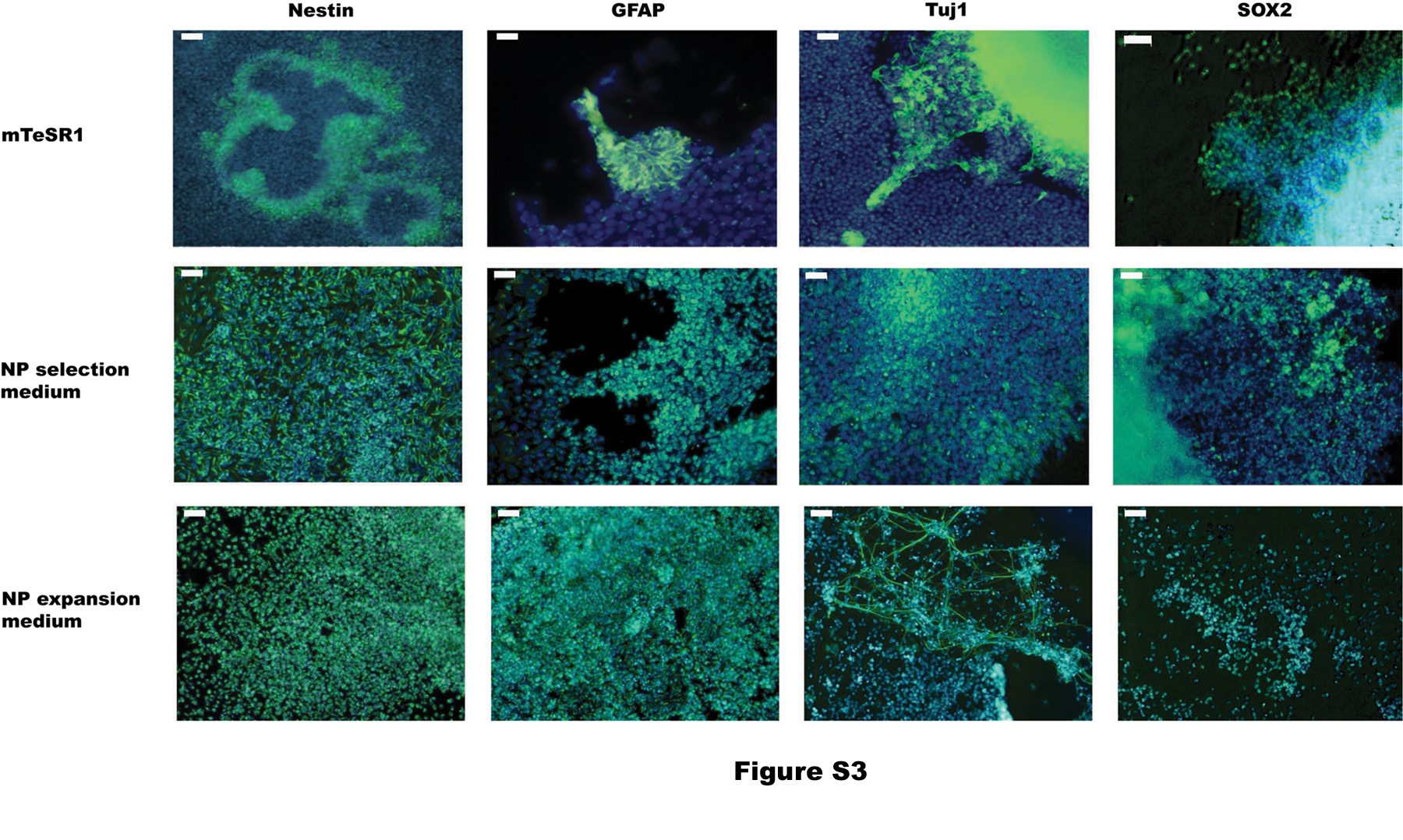

Supplement: Figure S3 — Immunocytochemical analysis of iPS cells and their progeny in mTeSR1 medium, Neural Selection Medium and Neural Expansion Medium. Scale bar is 50 µm. (TIF) [file pone.0049700.s003.tif]

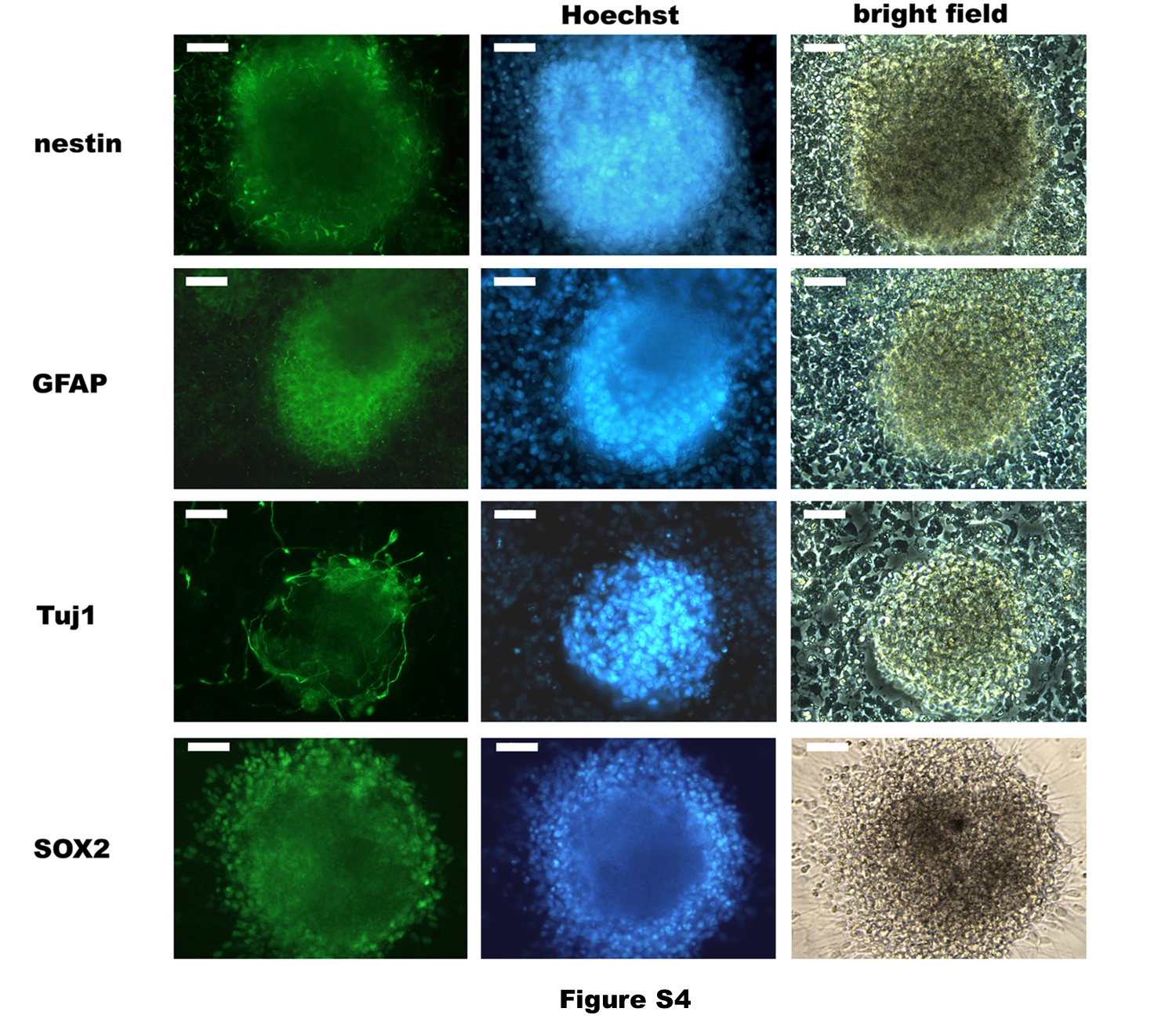

Supplement: Figure S4 — Immunocytochemical analysis of spherical cluster of cells generated during neural differentiation of iPS cells. Scale bar is 50 µm. (TIF) [file pone.0049700.s004.tif]

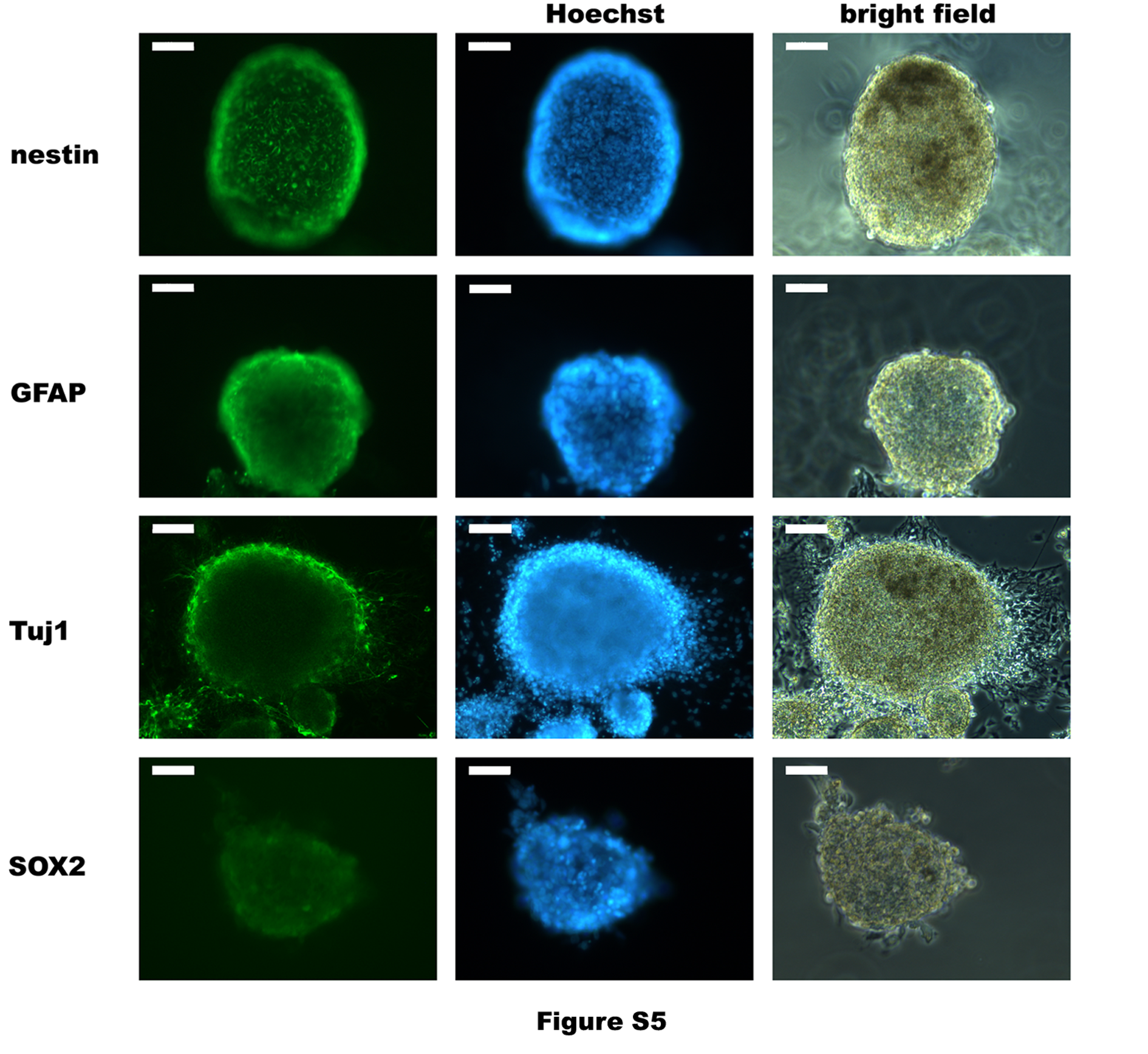

Supplement: Figure S5 — Immunocytochemical analysis of neurosphere-like structures. Scale bar is 50 µm. (TIF) [file pone.0049700.s005.tif]

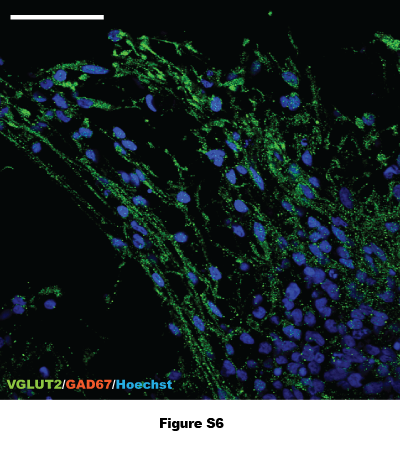

Supplement: Figure S6 — Immunocytochemical analysis of iPS-derived neurons. Scale bar is 50 µm. (TIF) [file pone.0049700.s006.tif]

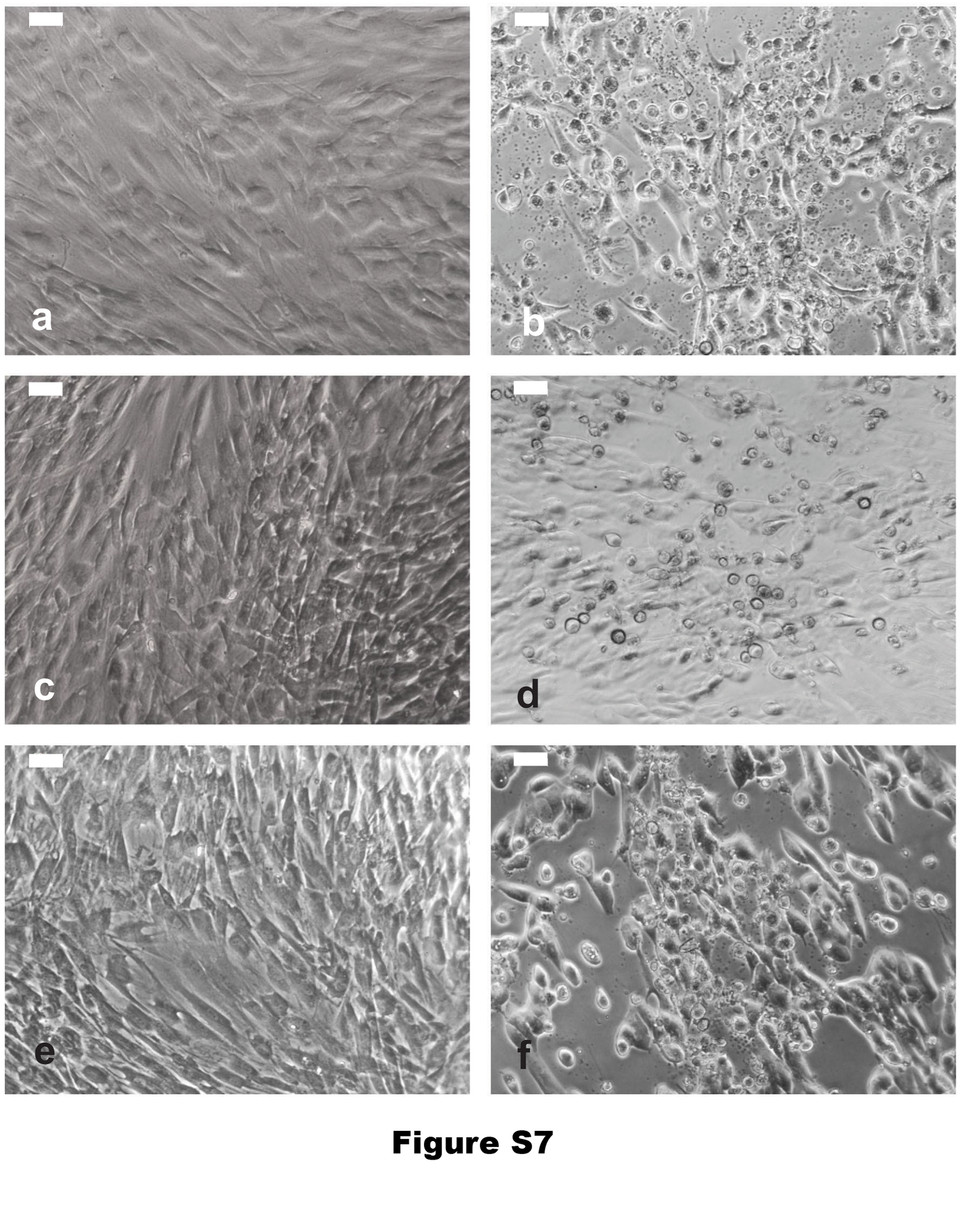

Supplement: Figure S7 — Evaluation of viral infection protocols. (a) Heat inactivation of HCMV was tested as an alternative form of mock infection. Heat-inactivated HCMV did not produce any CPE in comparison with (b) HCMV-infected fibroblasts. Media were collected from mock infected neural rosettes, mock infected neural progenitor cells, HCMV-infected neural rosettes, and HCMV-infected neural progenitor cells at day 15 p.i., cleared by centrifugation and diluted in the medium to infect fibroblasts. CPE was observed, starting from day 5 p.i., in fibroblast cultures exposed to the supernatant collected from HCMV-infected neural rosettes (d) and HCMV-infected neural progenitor cells (f), but not from mock infected neural rosettes (c) or mock infected neural progenitor cells (e). Scale bar is 50 µm. (TIF) [file pone.0049700.s007.tif]

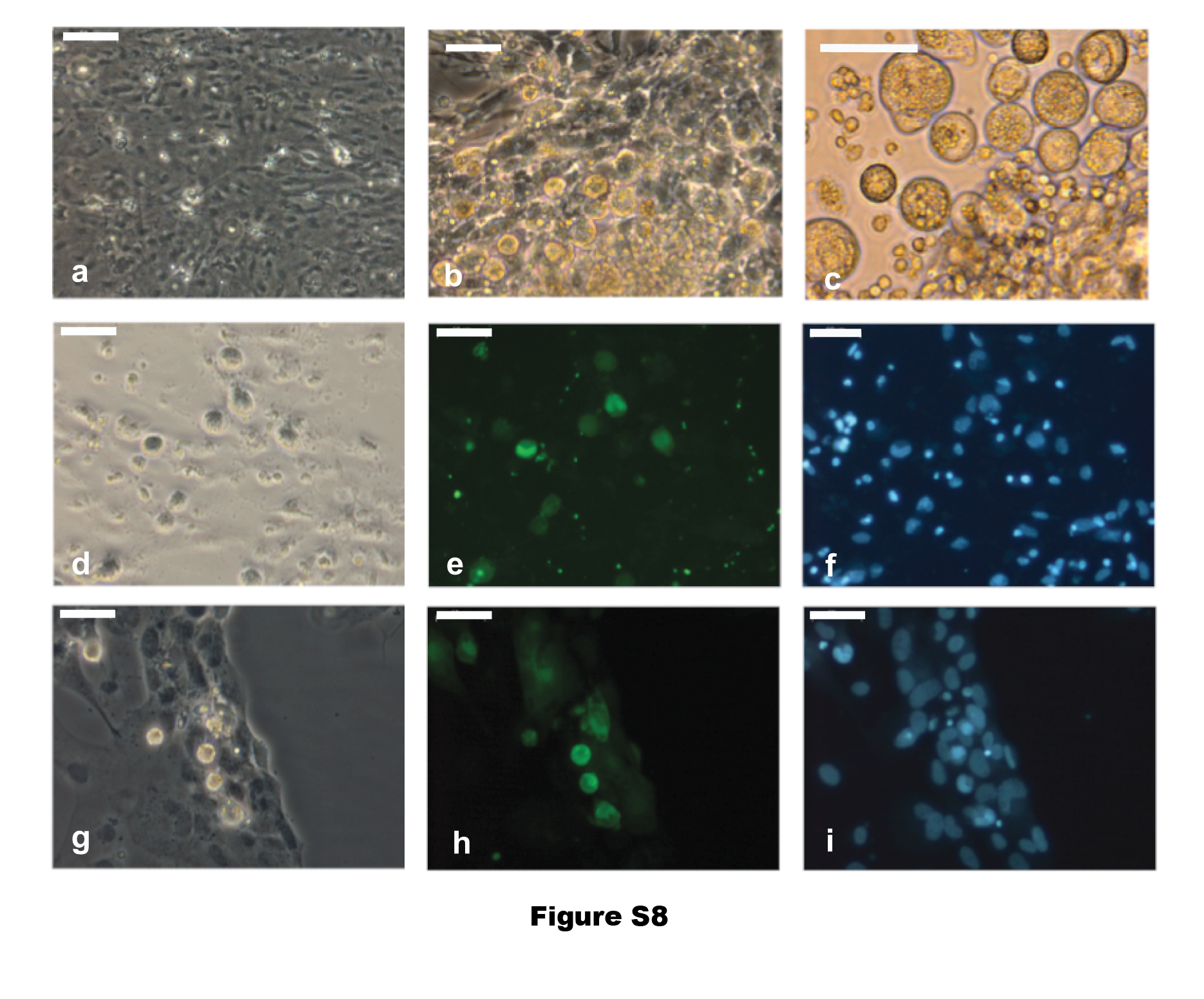

Supplement: Figure S8 — Immunocytochemical analysis of infected neural progenitor cells. Morphology of mock infected neural progenitor cells is depicted in (a). Degenerative change in infected cells is characterized by a round morphology with increased size (b, d, g), and detachment of cells from the surface of the dish (c). Expression of CMV immediate early gene (e) and nestin (h) in infected neural progenitor cells showing CPE. Cells were counterstained with Hoechst (f, i). Scale bar is 50 µm. (TIF) [file pone.0049700.s008.tif]

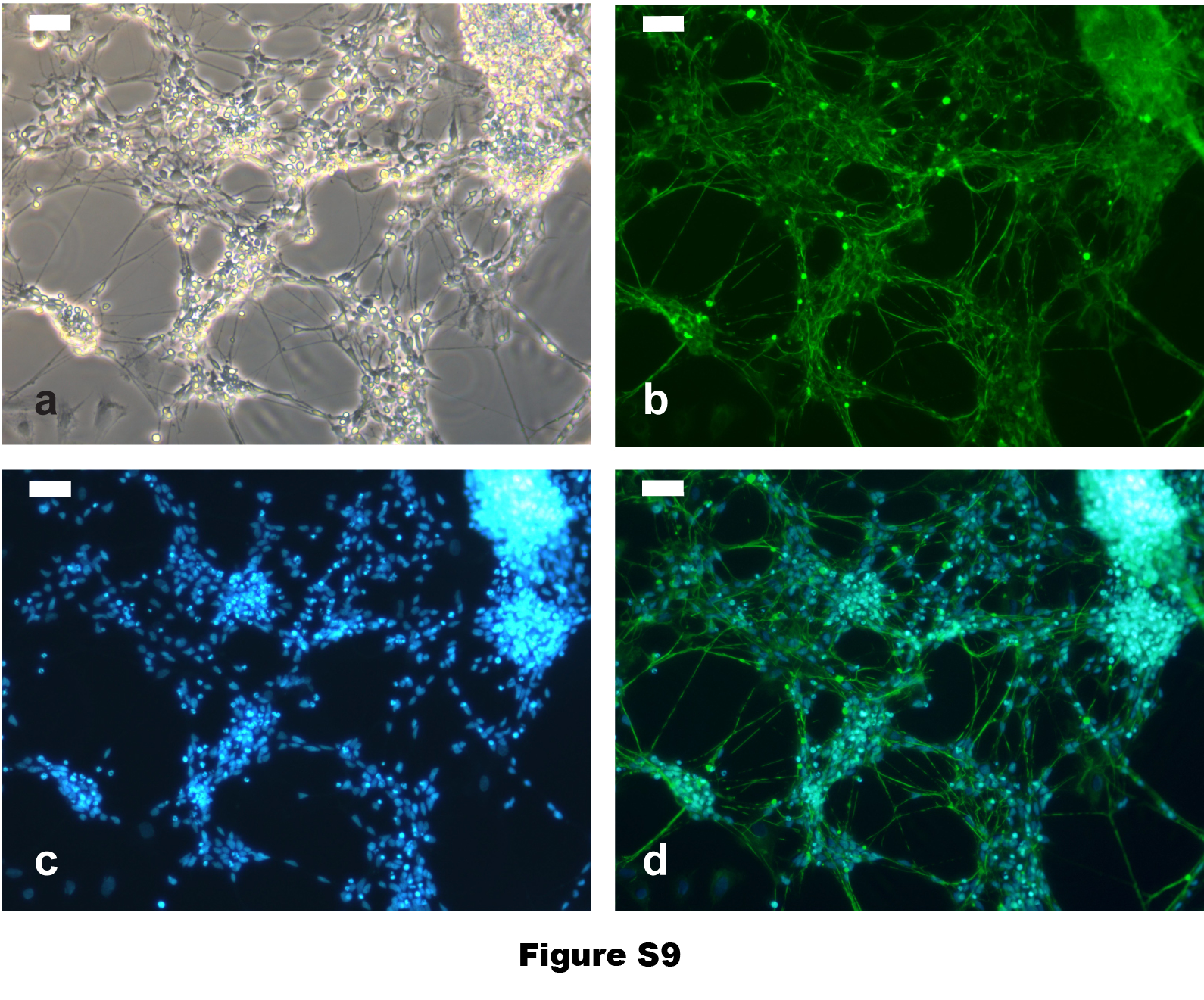

Supplement: Figure S9 — Tuj1 immunostainig of neuron-enriched cultures. (a) bright-field, (b) Tuj1, (c) Hoechst, (d) merge. Scale bar is 50 µm. (TIF) [file pone.0049700.s009.tif]

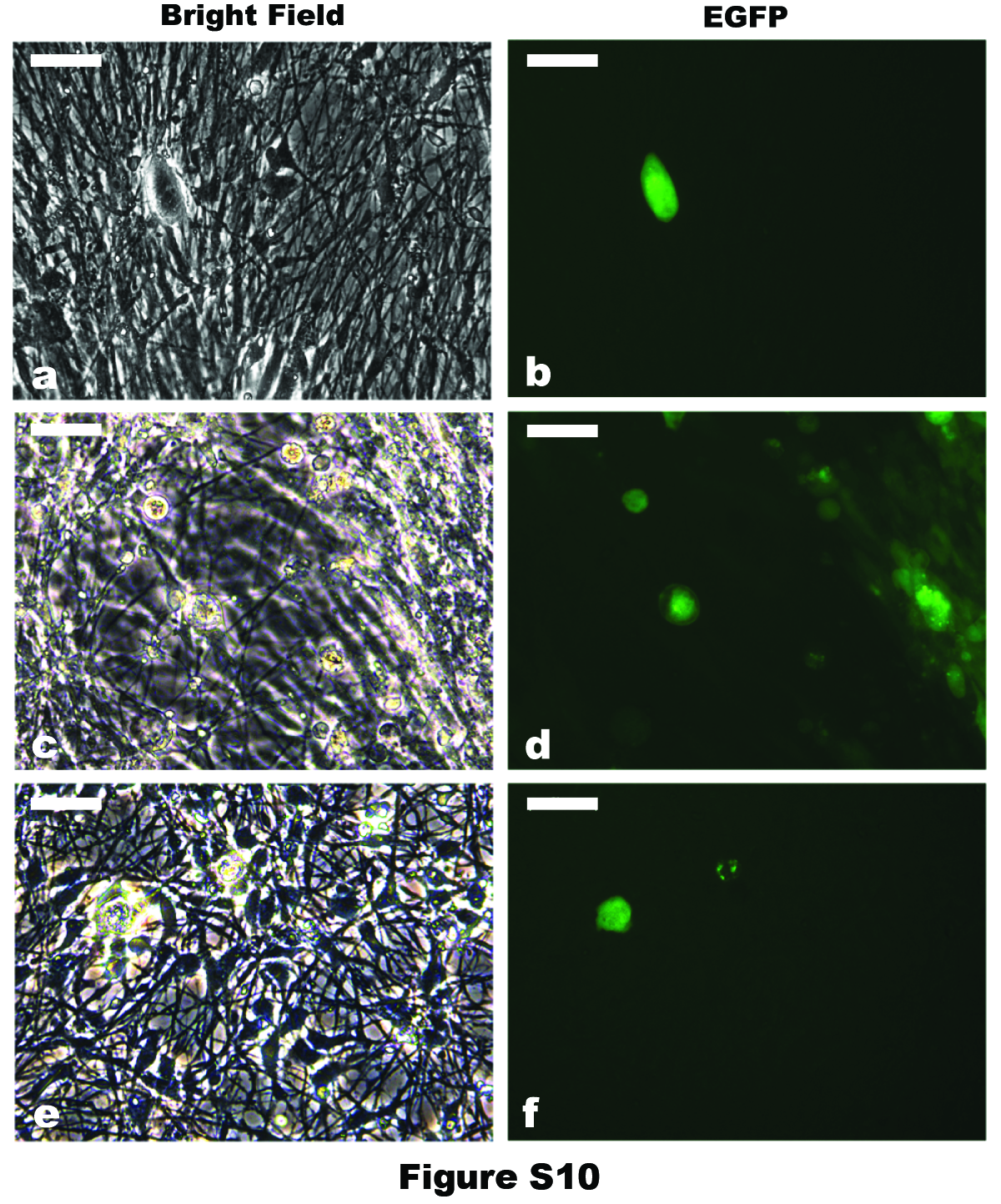

Supplement: Figure S10 — Infection of neuron-enriched cultures with UL32-EGFP-HCMV-TB40 strain. This recombinant HCMV strain expresses EGFP under the control of tegument protein pUL32. Microphotographs of infected neuronal cultures at day 6 (a–b), day 11 (c–d) and day 21. Scale bar is 50 µm. (TIF) [file pone.0049700.s010.tif]

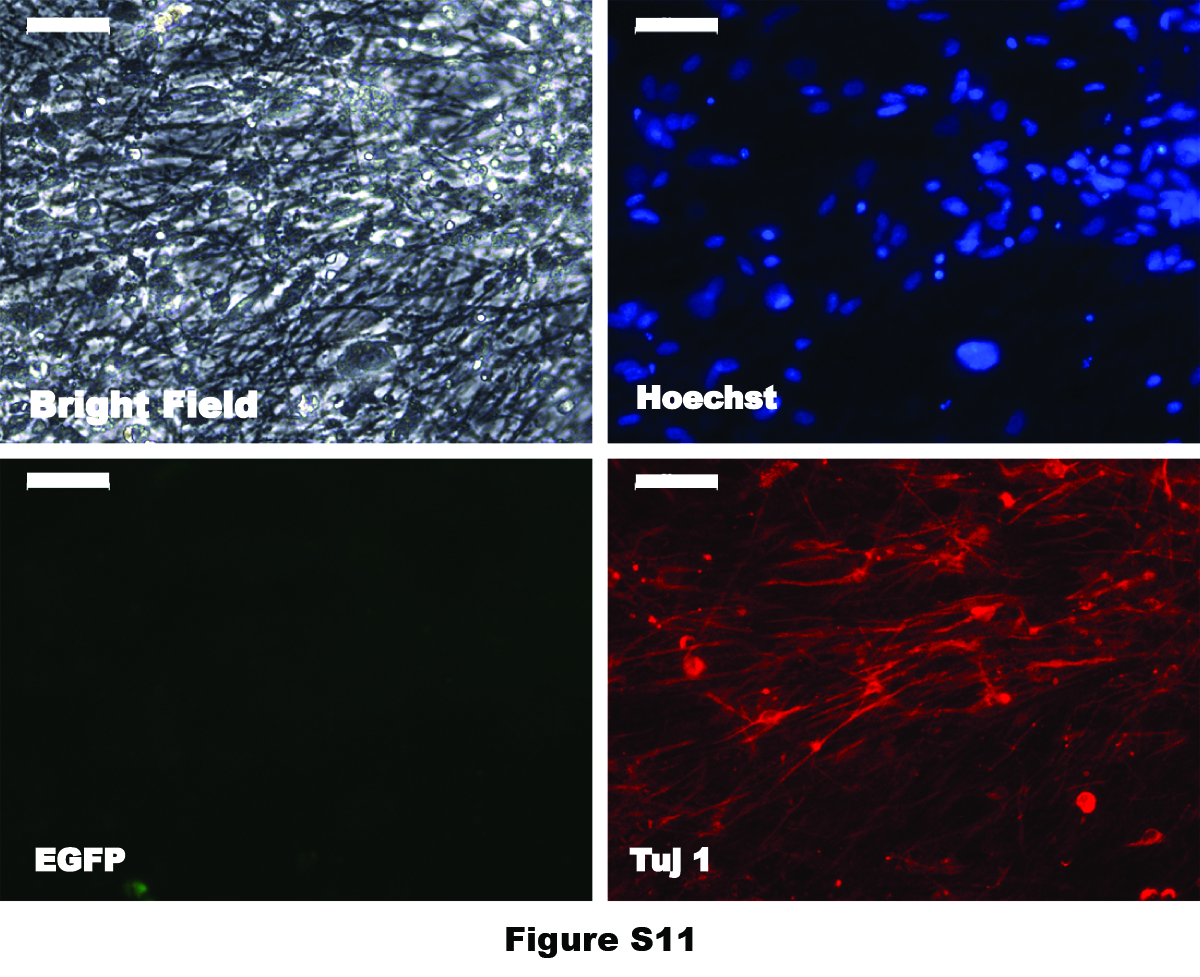

Supplement: Figure S11 — Mature neurons exposed to UL32-EGFP-HCMV-TB40 strain. Neuron-enriched cultures were infected with UL32-EGFP-HCMV-TB40 strain an MOI of 3. Expression of EGFP under the control of the HCMV UL32 gene was not detected in neurons staining with Tuj1. Nuclei counterstained with Hoechst. Scale bar is 50 µm. (TIF) [file pone.0049700.s011.tif]
