# Supplementary material for: Human Induced Pluripotent Stem Cell-Derived Models to Investigate Human Cytomegalovirus Infection in Neural Cells
Source: PLoS One. 2012 Nov 27;7(11):e49700. doi: 10.1371/journal.pone.0049700 (PMC3507916; doi:10.1371/journal.pone.0049700)
Supplement: Supporting Information S1 — (DOCX) [file pone.0049700.s012.docx]

**Supporting Information S1**

**Neural differentiation of iPS cells.**

We derived neurosphere-like structures directly from iPS cells. This strategy is based on our earlier observation that iPS cells (Figure 1a) adapted to mTeSR1 medium express nestin, GFAP, ß-tubulin III, and Sox2 when cultured for one week in NP Selection medium (Figure S3). The number of cells expressing the neural progenitor cell markers increases when the NP Selection medium is replaced with NP Expansion medium (Figure S3). After 3-5 days in NP expansion medium, the cell culture is characterized by the presence of spherical cluster of cells (Figure 1b) and neural rosettes (Figure 1c). The spherical clusters of cells also express ß-tubulin III, nestin, GFAP, and Sox2 (Figure S4).

To generate neurosphere-like structures (Figure 1d), neural rosettes and spherical cluster of cells were manually dissected into cellular aggregates after 7 days in NP Expansion medium and cultured in low attachment plates. After overnight incubation, cells aggregates rounded up into free-floating spheres resembling the neurospheres generated by culturing human sub-ventricular zone from postmortem brains^1^. Here, we refer to such cells as ‘neurosphere-like’ structures as we do not know if they share all the characteristics and differentiation potential of neuropsheres. The immunostaining of the neurosphere-like structures for nestin, ß-tubulin III, GFAP, and Sox2 is shown in Figure S5. Neurosphere-like structures were dissociated to single cells and expanded as monolayers of NPCs in NP expansion medium (Figure 1e) or cultured in neurobasal medium for neural differentiation (Figure 1f). Figure 1g-h shows the immunostaining of iPS-derived neurons for ß-tubulin III and MAP2. Significant increase of glutamate-mediated Ca2+ influx was observed in the neuronal cells (Figure 1j), but not in the NPC cells (Figure 1i), suggesting that the former are functionally active.

**Detailed materials and methods for neural differentiation of iPS cells.**

**Derivation of neurosphere-like structures from iPS cells**

iPS cells were cultured in Neural precursor (NP) selection medium (Dulbecco’s minimum essential medium/F12 medium supplemented with 0.5% N2, 1 mM L-glutamine, 1% nonessential amino acids, 50 U/ml penicillin, 50 µg/ml streptomycin and 0.1 mM 2-mercaptoethanol). Medium was changed every other day. After 7 days, the NP selection medium was replaced with Neural precursor (NP) expansion medium **(**Dulbecco’s minimum essential medium/F12 medium supplemented with 1% N2, bFGF (20 ng ml^-1^), 1 mM L-glutamine, 1% nonessential amino acids, 50 U ml^-1^ penicillin, 50 µg ml^-1^ streptomycin and 0.1 mM 2-mercaptoethanol) [1].

The medium was changed every other day until the appearance of either spherical cluster of cells and neural rosettes. To generate neurosphere-like structures, neural rosettes and the spherical cluster of cells were dissected manually and transferred into non-adherent 6-well plates (Costar) in NP expansion medium.

**Monolayer cultures of neural progenitor cell cultures**

Neurosphere-like structures were incubated with accutase for 5 minutes and then dissociated to single cells by pipetting them repeatedly. Cells were then transferred into matrigel-coated plates and cultured with NP expansion medium.

**Neuronal differentiation of neural progenitor cells.**

Neural progenitor cells (NPCs) were dissociated with accutase and plated onto matrigel-coated or fibronectin-coated 6-well plates in NP expansion medium. On the next day, NP expansion medium was changed to Neurobasal medium (Neurobasal medium supplemented with 1%B-27 and BDNF (10 ng ml^-1^). Under these conditions, NPCs differentiated in glutamatergic neurons. No GABAergic neurons were detected after 45 days of differentiation (Figure S6).

**Derivation of neuron-enriched cultures.**

Semi-confluent monolayer cultures of NPCs were cultured with Neurobasal medium. After 10 days, cells were dissociated using accutase (StemPro). Single cells were collected by centrifugation, washed twice with Phosphate Buffered Saline (PBS) and resuspended at a density of 1x10^5^/ml in neurobasal medium. The cell suspension was seeded on fibronectin coated 6-well plates. After 6 minutes, the cell suspension was removed from the plate and neuronal cells attached to the surface of the fibronectin-coated surface were cultured for 4 weeks in neurobasal medium. Half of the medium was refreshed every other day.

**REFERENCES**

1. Cho MS, Hwang DY, Kim DW (2008) Efficient derivation of functional dopaminergic neurons from human embryonic stem cells on a large scale. Nat Protoc 3:1888-94.
